# Ceruloplasmin Is a Novel Adipokine Which Is Overexpressed in Adipose Tissue of Obese Subjects and in Obesity-Associated Cancer Cells

**DOI:** 10.1371/journal.pone.0080274

**Published:** 2014-03-27

**Authors:** Erik Arner, Alistair R. R. Forrest, Anna Ehrlund, Niklas Mejhert, Masayoshi Itoh, Hideya Kawaji, Timo Lassmann, Jurga Laurencikiene, Mikael Rydén, Peter Arner, and the FANTOM consortium

**Affiliations:** 1 RIKEN Center for Life Science Technologies, Division of Genomic Technologies, Yokohama, Kanagawa, Japan; 2 Department of Medicine, Karolinska Institutet at Karolinska University Hospital, Huddinge, Huddinge, Sweden; 3 RIKEN Preventive Medicine and Diagnosis Innovation Program, Wako, Saitama, Japan; 4 RIKEN Omics Science Center, Yokohama, Kanagawa, Japan; Harbin Institute of Technology, China

## Abstract

Obesity confers an increased risk of developing specific cancer forms. Although the mechanisms are unclear, increased fat cell secretion of specific proteins (adipokines) may promote/facilitate development of malignant tumors in obesity via cross-talk between adipose tissue(s) and the tissues prone to develop cancer among obese. We searched for novel adipokines that were overexpressed in adipose tissue of obese subjects as well as in tumor cells derived from cancers commonly associated with obesity. For this purpose expression data from human adipose tissue of obese and non-obese as well as from a large panel of human cancer cell lines and corresponding primary cells and tissues were explored. We found expression of ceruloplasmin to be the most enriched in obesity-associated cancer cells. This gene was also significantly up-regulated in adipose tissue of obese subjects. Ceruloplasmin is the body's main copper carrier and is involved in angiogenesis. We demonstrate that ceruloplasmin is a novel adipokine, which is produced and secreted at increased rates in obesity. In the obese state, adipose tissue contributed markedly (up to 22%) to the total circulating protein level. In summary, we have through bioinformatic screening identified ceruloplasmin as a novel adipokine with increased expression in adipose tissue of obese subjects as well as in cells from obesity-associated cancers. Whether there is a causal relationship between adipose overexpression of ceruloplasmin and cancer development in obesity cannot be answered by these cross-sectional comparisons.

## Introduction

There is a worldwide increase in obesity prevalence and obesity and its co-morbidities have become a major health problem in most countries [1]. Obesity-associated cancer has recently gained much attention. The World Cancer Research Fund concluded in 2007 that obesity is convincingly associated with increased risk of pancreatic, (postmenopausal) breast, endometrial and renal cancer [2]. These conclusions were subsequently confirmed and extended in a recent meta-analysis of prospective studies assessing cancer risks associated with ≥5 kg/m^2^ increases in body mass index (BMI) [3]. The strongest risk (odds ratio ≥1.30) was observed for renal cancer and oesophagal adenocarcinoma in both genders, for thyroid cancer in men and for endometrial and gall bladder cancer in women.

The mechanisms linking obesity to cancer are poorly understood although several theories have been put forward [4], [5], [6], [7], [8]. Given that adipose tissue constitutes the body's largest endocrine organ, secreting hundreds of peptide signals collectively termed adipokines, and that adipokine secretion is significantly disturbed in obesity, an attractive hypothesis is that adipose-derived signals to other tissues could be involved in cancer development. The two most thoroughly studied adipokines, adiponectin and leptin, have in fact been linked to the development of malignant tumors through their action on cognate receptors affecting insulin sensitivity and/or activating cancer-associated signaling [4]. Furthermore, several adipokines are growth factors which could serve as direct signals to tumors [9]. These as well as other, not yet discovered adipokines, may therefore have cancer-promoting properties.

In this study, we aimed at finding novel human adipokines that may be associated with obesity-associated cancers. To this end, we extracted data from the novel FANTOM5 expression atlas, which provides an unprecedented coverage of human tissues, primary cells and cancer cell lines [10]. We focused on the cancer cell lines derived from malignant tumors having the strongest association with obesity (odds ratio ≥1.3 in either sex) according to Renehan et al [3]. Putative novel adipokines of potential relevance for obesity-associated cancers were identified by comparing the transcriptome enriched in both cancer cells and obese adipose tissue. This was done by combining the FANTOM5 expression atlas data with a recently published study of gene expression in adipose tissue from a large cohort of obese and non-obese women [11].

This work is part of the FANTOM5 project. Data downloads, genomic tools and co-published manuscripts are summarized here http://fantom.gsc.riken.jp/5/.

## Methods

### Subjects and adipose tissue

All subjects were informed in detail about the study and written informed consent was obtained. The studies were approved by the Committee of Ethics at Karolinska Institutet, Stockholm, Sweden. Three different cohorts of subjects were used in the study. In the first cohort, described previously [11], subcutaneous adipose tissue was obtained from 26 non-obese and 30 obese women. Obesity was defined as BMI 30 kg/m^2^ or above. RNA was extracted and subjected to global expression microarray as described [11]. The gene expression data have been deposited in the National Center for Biotechnology Gene Expression Omnibus (accession number GSE25402). The protocol number from the Committee of Ethics is 592/03 (approved 1^st^ of December 2003).

In the second cohort, subcutaneous adipose tissue was obtained by cosmetic liposuction and used to experimentally identify novel adipokines. Nine women were investigated. All were non-obese and healthy according to self-report. Values (mean ±SD) for age and BMI were 45±13 years and 27.4±1.2 kg/m^2^. The tissue was subjected to collagenase digestion and the stroma-vascular portion was differentiated into adipocytes in culture as described [11]. For protein determination in conditioned media, aliquots were removed 4, 8 and 12 days after the start of differentiation. The protocol number from the Committee of Ethics is 2009/1881-31/1 (approved 14^th^ of January 2010).

In the third cohort, adipose tissue secretion of proteins and their plasma levels were determined. Twenty obese (age 46±6 years, mean±SD) and 19 non-obese healthy women (age 47±9 years) were investigated in the morning after an overnight fast. The obese were scheduled for bariatric surgery. Seven were healthy, 10 had hypertension and three had type 2 diabetes treated with oral agents. Total body fat was determined by dual x-ray absorptiometry (62±10 kg in obese and 24±8 kg in non-obese). Thereafter, a venous plasma sample was obtained for determination of circulating protein levels. Finally, adipose tissue was obtained by a subcutaneous abdominal biopsy as described [11], [12]. Part of the adipose tissue sample was subjected to *in vitro* incubation for 2 h and conditioned medium was used for protein determination as described [12]. The other part was subjected to collagenase treatment and isolated fat cells were obtained for the determination of fat cell size and volume as described [13]. Protein secretion was related to the number of fat cells in the incubated tissue sample and to the total amount of body fat. In the latter case, protein secretion was related to the amount of lipids in the incubated tissue and this value was multiplied by the total body fat weight as described [12]. The protocol number from the Committee of Ethics is 2005/1441-32 (approved 1^st^ of February 2006).

### Protein measures

Ceruloplasmin and osteopontin were measured by commercial ELISA kits (Abcam, Cambridge, UK).

### Adipose contribution to total ceruloplasmin production

In order to estimate the contribution of adipose tissue-derived ceruloplasmin to the circulation, the following assumptions were made: (a) ceruloplasmin secretion from adipose tissue incubates *in vitro* is similar in different adipose regions and reflects *in vivo* secretion. (b) ceruloplasmin is evenly distributed in the water space which is 0.530 l/kg bodyweight in non-obese and 0.465 l/kg bodyweight in obese subjects [14]. (c) the plasma level of ceruloplasmin in the morning reflects overall 24 hour levels and the half-life of the protein is 5.5 days [15]. Based on these assumptions, total ceruloplasmin production per hour was estimated as: (measured plasma level x 5.5 x total body water) divided by 24. Total adipose secretion per hour is measured as secretion per lipid weight of adipose tissue per hour times total body fat. From these values the percentage of adipose tissue contribution to ceruloplasmin production can be calculated.

### Cancer and control samples

Based on Renehan et al [3], we identified the following cancer cell lines in the FANTOM 5 data set as being derived from tumor types associated to obesity and having an odds ratio ≥1.30 in men, women or both. Uterus: endometrial stromal sarcoma cell line (OMC-9), endometrial carcinoma cell line (OMC-2), endometrioid adenocarcinoma cell line (JHUEM-1), clear cell carcinoma cell line (TEN); Kidney: renal cell carcinoma cell line (OS-RC-2), renal cell carcinoma cell line (TUHR10TKB); Gall bladder: gall bladder carcinoma cell line (TGBC2TKB), gall bladder carcinoma cell line (TGBC14TKB); Thyroid: thyroid carcinoma cell line (TCO-1), thyroid carcinoma cell line (KHM-5 M), papillary adenocarcinoma cell line (8505C). As control samples we used the following tissue and primary cell samples. Uterus: adult and fetal tissue; Kidney: adult and fetal tissue, Renal Cortical Epithelial Cells (two donors), Renal Epithelial Cells (three donors), Renal Glomerular Endothelial Cells (three donors), Renal Mesangial Cells (three donors), Renal Proximal Tubular Epithelial Cell (three donors); Gall bladder: adult tissue; Thyroid: adult and fetal tissue.

### Identification of novel cancer-related adipokine candidates

Gene expression in the FANTOM5 database was calculated as the normalized tag count (tags per million, TPM) in a 1 kb window around RefSeq [16] transcription start sites. Genes were identified as candidates if they were expressed at a functionally significant level (here defined as 10 TPM or higher) and enriched at least two-fold in the above listed cancer cell lines as compared to all cell lines available in FANTOM5, while not being enriched among the corresponding tissues or primary cells. Enrichment was computed as described in [17] with p-values corrected for multiple testing using the Benjamini-Hochberg method [18]. Candidate genes were retained if they were significantly up-regulated at 5% FDR in the adipose tissue of obese subjects according to a previous study [11]. Using the FANTOM5 expression database, we required at least 10 TPM CAGE gene expression across a receptor locus in one or more obesity associated cancer cell lines in order to retain it for further investigation.

### Statistical analysis

Values are mean ± standard error. They were compared by analysis of variance (repeated measure), unpaired t-test and linear regression analysis.

## Results

**Figure 1:**
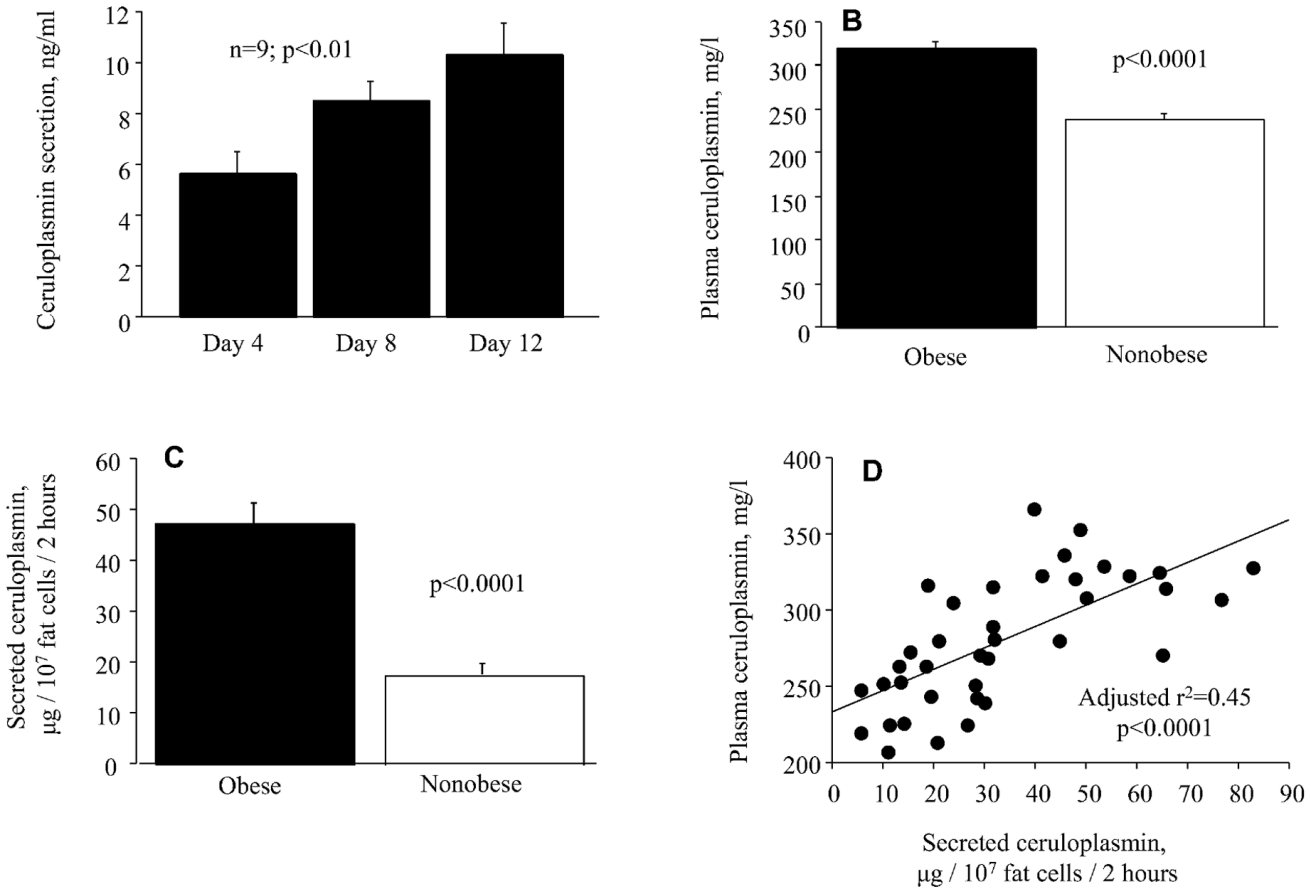
Secretion of ceruloplasmin from fat cells. A. Secretion during differentiation of progenitor cells to fat cells. Data are analysed by ANOVA, repeated measures. B and C. Influence of obesity on plasma levels and secretion from adipose tissue as analyzed with unpaired t-test. D. Relationship between secreted ceruloplasmin and its plasma levels as analyzed with linear regression.

Initial analysis of the FANTOM 5 data base [10] and the published adipose tissue array [11] identified 700 genes which were significantly expressed (≥10 TPM) and at least two-fold enriched in a cancer cell line of interest but not enriched in the corresponding tissue or primary cells, and up-regulated in the adipose tissue of obese. It was not feasible to perform a detailed analysis of all identified genes or to determine which of them that were actually secreted from fat cells. Therefore, we further narrowed the list by restricting expression in the relevant cancer cell line to ≥100 TPM (a high expression level), at least five-fold enrichment in the cancer cell line compared with corresponding healthy tissue/cell, and at least two-fold up-regulation in adipose tissue in obesity. Only two genes met these stricter criteria, namely ceruloplasmin (geneID: *CP*) and osteopontin (geneID: *SPP1*). Surprisingly, both of them encoded proteins present in the circulation and were therefore considered potential adipokines. We investigated whether ceruloplasmin and osteopontin were actively secreted from fat cells by determining whether there was a time-dependent release into the media. While osteopontin was not detectable in the incubation medium (graph not shown), ceruloplasmin was indeed released in a time dependent fashion in differentiating adipocytes (Fig. 1a). Thus, although osteopontin mRNA is expressed in adipose tissue according to microarray and is upregulated in obesity [11], the encoded protein does not appear to be secreted from fat cells implying that osteopontin is not an adipokine. The influence of obesity on plasma levels and secretion of ceruloplasmin from subcutaneous adipose tissue was also investigated (Fig 1b-d). Plasma levels and adipose secretion were significantly increased in samples from obese subjects. There was a strong positive relationship between circulating and adipose-secreted ceruloplasmin. In fact, the latter explained 45% of the inter-individual variation in plasma ceruloplasmin (adjusted r^2^), which was independent of BMI (partial r = 0.36; p = 0.026) or body fat (partial r = 0.44; p = 0.007). The contribution of adipose tissue to total ceruloplasmin production was determined as described in Methods and was estimated to be 13±5% in non-obese and 22±6% in obese (p<0.0001).

**Figure 2:**
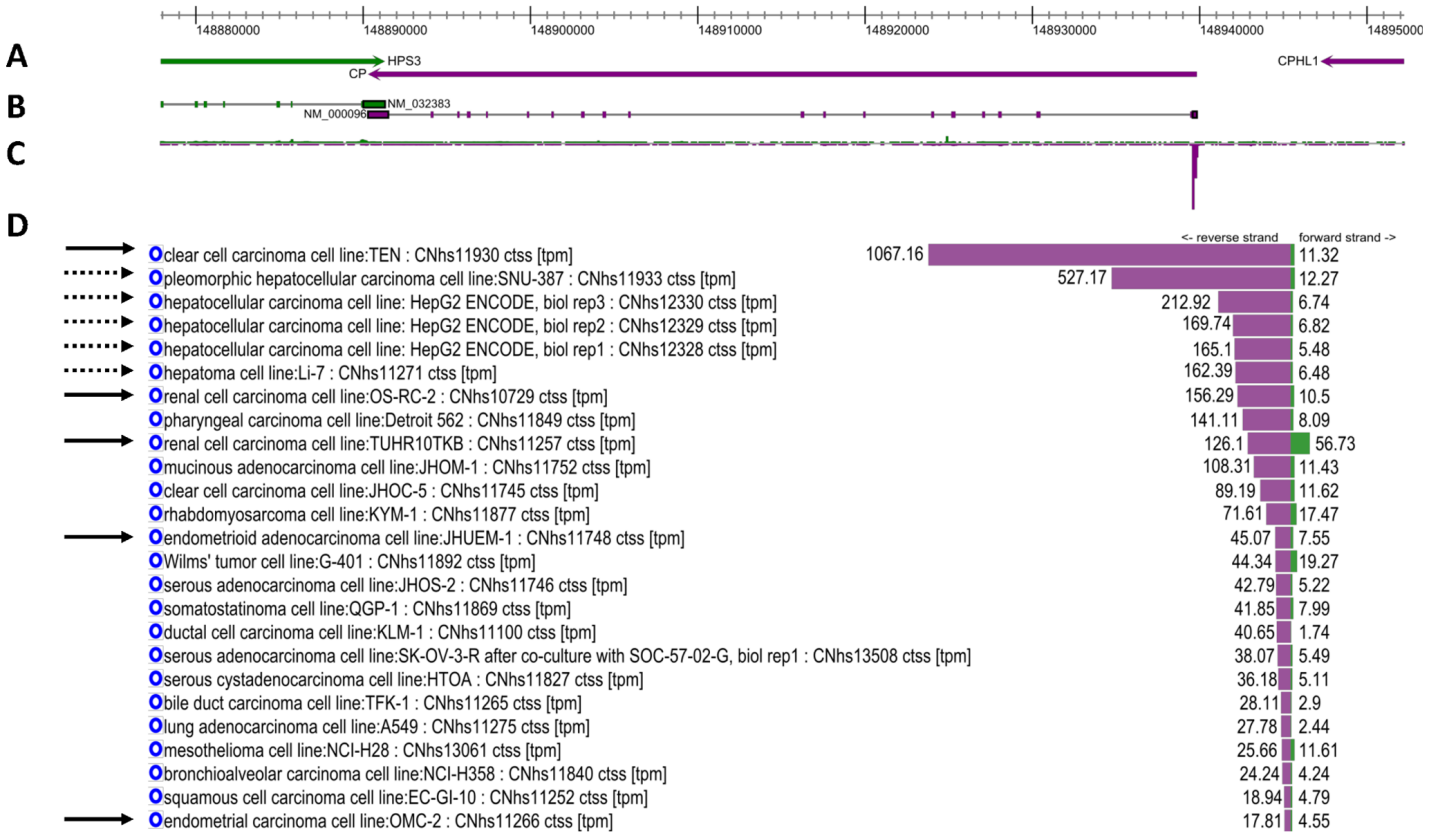
View of the ceruloplasmin (CP) locus in the human genome. A. CP is located on on the negative (purple) strand of chromosome 3 at position 3q23-q25, flanked by HPS3 on the positive (green) strand and CPHL1 on the negative strand. B. RefSeq mRNA models of the locus. C. Promoter activity signal distribution as measured by CAGE in the locus. The majority of expression comes from the 5′ end of CP. D. Expression (tags per million, TPM) across the locus for the cell lines present in FANTOM5. Cell lines with expression above 10 TPM are shown here; in total 269 cell lines were profiled. Obesity associated cell lines are indicated by black (O.R. > = 1.30) and gray (O.R. > = 1.20) arrows.

We further investigated the expression pattern of *CP* in human cancer cells. Figure 2 shows the genomic location of *CP* (Fig 2a, b), the distribution of promoter activity as measured by cap analysis gene expression (CAGE, Fig 2c) and *CP* expression across a wide panel of cancer cell types sorted by expression level (Fig 2d). Notably, the cancer cell line with the highest expression of *CP* is a clear cell carcinoma from endometrium (Fig 2d, top solid arrow). Other obesity-associated cancers (renal, endometrial) also display relatively high *CP* expression (fig 2d, solid arrows). Hepatic cancer was not included in this study because it did not meet our criterion of obesity/cancer association odds ratio of 1.30 or more; nevertheless it is interesting to note that it is associated with obesity in men when using slightly less strict criteria (odds ratio = 1.24 in reference[3]) and that several liver cancer cell lines are among those with the highest *CP* expression (fig 2d, broken arrows). Among the nine cancer cell lines with the highest *CP* expression, seven were associated with obesity.

## Discussion

In this study we took a systematic approach to identify novel adipokines that were associated with obesity and obesity-related cancers by comparing the expression of all obesity-induced genes in adipose tissue with genes enriched in all cancer cell lines included in the FANTOM5 study. Using rather stringent selection criteria, only two genes scored very high in this comparison. In theory, these genes could have encoded secreted as well as non-secreted proteins with any type of function. However, it turned out that both genes encoded circulatory proteins, namely osteopontin and ceruloplasmin. Osteopontin could not be defined as an adipokine (protein secreted from fat cells) according to our findings. In contrast, ceruloplasmin fulfilled the criteria for a true adipokine, i.e. it is secreted in a time-dependent fashion. Moreover, since it is not included in the published secretome for human fat cells, we consider it a novel adipokine [9]. However, our study cannot establish whether there is a casual relationship between ceruloplasmin and obesity-associated cancers, and further studies are needed to determine to what extent, if any, ceruloplasmin derived from adipocytes or tumor cells plays a functional role in the growth of cancer tissue. Nevertheless, we can state that *CP* is overexpressed in obese adipose tissue and in cancer cells associated with obesity.

Ceruloplasmin in the circulation is believed to be primarily produced by the liver [15], [19]. However, the present data suggest a hitherto unknown contributing role of adipose tissue. In line with earlier findings [20], [21], the circulating levels were increased in obesity. Furthermore, adipose secretion was markedly increased in obese versus non-obese subjects. Our estimates suggest that adipose tissue in obesity contributes with up to 22% to the circulating levels of ceruloplasmin and this secretion explained as much as 45% of the inter-individual variation in plasma ceruloplasmin in both non obese and obese subjects. The relationship between circulating and adipose-secreted ceruloplasmin was independent of BMI or body fat mass. It should be stressed that the estimated *in vivo* values are calculated based on *in vitro* results which may be different from those *in vivo*. Moreover, the results may be gender biased since the adipose tissue microarray data used herein was from women while assumptions on ceruloplasmin distribution were partly based on a previous study in men [14]. In fact, although we measured ceruloplasmin secretion in adipose tissue from women, we used male data for total body water [14] since the latter values were obtained using a state-of-the-art technique. However, total body water values for women measured with the less accurate biompedance method are comparable with those used in our present calculations, namely 0.527±0.04, 0.472±.03, and 0.426±0.028 l/kg body weight in lean, overweight and obese females, respectively [22].

Finally, our study examined only subcutaneous adipose tissue. Since expression and secretion of some adipokines are differentially regulated in different fat depots [23], we do not know whether ceruloplasmin expressed and released from other depots may display other correlations with obesity. On the other hand, subcutaneous adipose tissue is by far the body's largest fat depot and therefore quantitatively the most important determinant of circulating adipokine levels.

In summary, by bioinformatically performing a systematic screening for genes overexpressed in obesity associated cancers, this study identified ceruloplasmin as a novel human adipokine. Its secretion and expression are increased in obesity and adipose ceruloplasmin is a major contributor to the circulating ceruloplasmin level.

## Supporting Information

Table S1 List of FANTOM5 consortium members.
(DOCX)
