# Supplementary material for: Ceruloplasmin Is a Novel Adipokine Which Is Overexpressed in Adipose Tissue of Obese Subjects and in Obesity-Associated Cancer Cells
Source: PLoS One. 2014 Mar 27;9(3):e80274. doi: 10.1371/journal.pone.0080274 (PMC3968011; doi:10.1371/journal.pone.0080274)
Supplement: Table S1 — List of FANTOM5 consortium members. (DOCX) [file pone.0080274.s001.docx]

Alistair R.R. Forrest^1,98^, Hideya Kawaji^1,97,98^, Michael Rehli^2^, J. Kenneth Baillie^3^, Michiel J.L. de Hoon^1,98^, Vanja Haberle^4,5^, Timo Lassmann^1,98^, Ivan V. Kulakovskiy^6,7^, Marina Lizio^1,98^, Masayoshi Itoh^1,97,98^, Robin Andersson^8^, Christopher J. Mungall^9^, Terrence F. Meehan^10^, Sebastian Schmeier^11,96^, Nicolas Bertin^1,98^, Mette Jørgensen^8^, Emmanuel Dimont^12^, Erik Arner^1,98^, Christian Schmidl^2^, Ulf Schaefer^11^, Yulia A. Medvedeva^11^, Charles Plessy^1,98^, Morana Vitezic^1,14^, Jessica Severin^1,98^, Colin A. Semple^13^, Yuri Ishizu^1,98^, Margherita Francescatto^15,16^, Intikhab Alam^11^, Davide Albanese^17^, Gabriel M. Altschuler^12^, John A.C. Archer^11^, Peter Arner^18^, Magda Babina^19^, Sarah Baker^13^, Piotr J. Balwierz^20^, Anthony G. Beckhouse^21,22^, Swati Pradhan-Bhatt^23^, Judith A. Blake^24^, Antje Blumenthal^22,25^, Beatrice Bodega^26^, Alessandro Bonetti^1^, James Briggs^21^, Frank Brombacher^27,28^, A. Maxwell Burroughs^1^, Andrea Califano^29,30,31,32^, Carlo V. Cannistraci^33,34^, Daniel Carbajo^75^, Yun Chen^8^, Marco Chierici^17^, Yari Ciani^35^, Hans C. Clevers^36,37,38^, Emiliano Dalla^35^, Carrie A. Davis^39^, Michael Detmar^41^, Alexander D. Diehl^42^, Taeko Dohi^43^, Finn Drabløs^44^, Albert S.B. Edge^45^, Matthias Edinger^2,99^, Karl Ekwall^46^, Mitsuhiro Endoh^47^, Hideki Enomoto^48^, Michela Fagiolini^49^, Lynsey Fairbairn^3^, Hai Fang^50^, Mary C. Farach-Carson^51^, Geoffrey J. Faulkner^52^, Alexander V. Favorov^7,53,54^, Malcolm E. Fisher^3^, Martin C. Frith^55^, Rie Fujita^56^, Shiro Fukuda^1^, Cesare Furlanello^17^, Masaaki Furuno^1,98^, Jun-ichi Furusawa^47,57^, Teunis B. Geijtenbeek^58^, Andrew Gibson^59^, Thomas Gingeras^39^, Daniel Goldowitz^60^, Julian Gough^50^, Sven Guhl^19^, Reto Guler^27,28^, Stefano Gustincich^61^, Thomas J. Ha^60^, Masahide Hamaguchi^62^, Mitsuko Hara^63^, Matthias Harbers^1^, Jayson Harshbarger^1,98^, Akira Hasegawa^1,98^, Yuki Hasegawa^1,98^, Takehiro Hashimoto^1^, Meenhard Herlyn^64^, Kelly J. Hitchens^21,22^, Shannan J. Ho Sui^12^, Oliver M. Hofmann^12^, Ilka Hoof^8^, Fumi Hori^1,98^, Lukasz Huminiecki^14^, Kei Iida^65^, Tomokatsu Ikawa^47^, Boris R. Jankovic^11^, Hui Jia^66^, Anagha Joshi^3^, Giuseppe Jurman^17^, Bogumil Kaczkowski^1,98^, Chieko Kai^67^, Kaoru Kaida^1,98^, Ai Kaiho^1^, Kazuhiro Kajiyama^1,98^, Mutsumi Kanamori-Katayama^1^, Artem S. Kasianov^7^, Takeya Kasukawa^98^, Shintaro Katayama^1^, Sachi Kato^1,98^, Shuji Kawaguchi^65^, Hiroshi Kawamoto^47^, Yuki I. Kawamura^43^, Tsugumi Kawashima^1,98^, Judith S. Kempfle^45^, Tony J. Kenna^25^, Juha Kere^46,68^, Levon M. Khachigian^69^, Toshio Kitamura^70^, S. Peter Klinken^71^, Alan J. Knox^72^, Miki Kojima^1,98^, Soichi Kojima^63^, Naoto Kondo^1,98^, Haruhiko Koseki^47^, Shigeo Koyasu^47,57^, Sarah Krampitz^41^, Atsutaka Kubosaki^1^, Andrew T. Kwon^1,98^, Jeroen F.J. Laros^59^, Weonju Lee^73^, Andreas Lennartsson^46^, Kang Li^8^, Berit Lilje^8^, Leonard Lipovich^66^, Alan Mackay-sim^74^, Ri-ichiroh Manabe^1,98^, Jessica C. Mar^75^, Benoit Marchand^11^, Anthony Mathelier^60^, Niklas Mejhert^18^, Alison Meynert^13^, Yosuke Mizuno^76^, David A. de Lima Morais^77^, Hiromasa Morikawa^62^, Mitsuru Morimoto^48^, Kazuyo Moro^47,57,78^, Efthymios Motakis^1,98^, Hozumi Motohashi^79^, Christine L. Mummery^80^, Mitsuyoshi Murata^1,98^, Sayaka Nagao-Sato^1^, Yutaka Nakachi^76,81^, Fumio Nakahara^70^, Toshiyuki Nakamura^67^, Yukio Nakamura^82^, Kenichi Nakazato^1^, Erik van Nimwegen^20^, Noriko Ninomiya^1^, Hiromi Nishiyori^1,98^, Shohei Noma^1,98^, Tadasuke Nozaki^83^, Soichi Ogishima^84^, Naganari Ohkura^62^, Hiroko Ohmiya^1,98^, Hiroshi Ohno^47^, Mitsuhiro Ohshima^85^, Mariko Okada-Hatakeyama^47^, Yasushi Okazaki^76,81^, Valerio Orlando^26^, Dmitry A. Ovchinnikov^21^, Arnab Pain^11,33^, Robert Passier^80^, Margaret Patrikakis^69^, Helena Persson^46^, Silvano Piazza^35^, James G.D. Prendergast^13^, Owen J.L. Rackham^50^, Jordan A. Ramilowski^1,98^, Mamoon Rashid^11,33^, Timothy Ravasi^33,34^, Patrizia Rizzu^15^, Marco Roncador^17^, Sugata Roy^1,98^, Morten B. Rye^44^, Eri Saijyo^1^, Antti Sajantila^86^, Akiko Saka^1^, Shimon Sakaguchi^62^, Mizuho Sakai^1,98^, Hiroki Sato^67^, Hironori Satoh^56^, Suzana Savvi^27,28^, Alka Saxena^1^, Claudio Schneider^35^, Erik A. Schultes^59^, Gundula G. Schulze-Tanzil^87^, Anita Schwegmann^27,28^, Thierry Sengstag^1^, Guojun Sheng^48^, Hisashi Shimoji^1^, Yishai Shimoni^32^, Jay W. Shin^1,98^, Christophe Simon^1,98^, Daisuke Sugiyama^88^, Takaaki Sugiyama^67^, Masanori Suzuki^1^, Rolf K. Swoboda^64^, Peter A.C. 't Hoen^59,89^, Michihira Tagami^1,98^, Naoko Takahashi^1,98^, Jun Takai^56^, Hiroshi Tanaka^84^, Hideki Tatsukawa^90^, Zuotian Tatum^59^, Mark Thompson^59^, Hiroo Toyoda^83^, Tetsuro Toyoda^65^, Eivind Valen^91^, Marc van de Wetering^36^, Linda M. van den Berg^58^, Roberto Verardo^35^, Dipti Vijayan^21,22^, Ilya E. Vorontsov^7^, Wyeth W. Wasserman^60^, Shoko Watanabe^1^, Christine A. Wells^21,22^, Louise N. Winteringham^71^, Ernst Wolvetang^21^, Emily J. Wood^66^, Yoko Yamaguchi^92^, Masayuki Yamamoto^56^, Misako Yoneda^67^, Yohei Yonekura^48^, Shigehiro Yoshida^1,98^, Suzan E. Zabierowski^93^, Peter G. Zhang^60^, Xiaobei Zhao^8^, Silvia Zucchelli^61^, Kim M. Summers^3^, Harukazu Suzuki^1,98^, Carsten O. Daub^1^, Jun Kawai^1,97^, Peter Heutink^15^, Winston Hide^12^, Tom C. Freeman^3^, Boris Lenhard^5,94^, Vladimir B. Bajic^11^, Martin S. Taylor^13^, Vsevolod J. Makeev^7,95^, Albin Sandelin^8^, David A. Hume^3^, Piero Carninci^1,98^, Yoshihide Hayashizaki^1,97^

^1^RIKEN Omics Science Center (OSC), 1-7-22 Suehiro-cho, Tsurumi-ku, Yokohama, 230-0045 Japan.

^2^Department of Internal Medicine III, University Hospital Regensburg, F.-J.-Strauss Allee 11, D-93042 Regensburg, Germany.

^3^The Roslin Institute and Royal (Dick) School of Veterinary Studies, University of Edinburgh, Easter Bush, Edinburgh, Midlothian, EH25 9RG Scotland, UK.

^4^Department of Biology, University of Bergen, Thormøhlensgate 53, NO-5006 Bergen, Norway.

^5^Faculty of Medicine, Institute of Clinical Sciences, MRC Clinical Sciences Centre, Imperial College London, Hammersmith Hospital Campus, London W12 0NN, UK.

^6^Laboratory of Bioinformatics and Systems Biology, Engelhardt Institute of Molecular Biology (EIMB), Vavilov str. 32, 119991 Moscow, Russia.

^7^Department of Computational Systems Biology, Vavilov Institute of General Genetics (VIGG), Gubkin str. 3, Moscow 119991, Russia.

^8^The Bioinformatics Centre, Department of Biology and BRIC, University of Copenhagen, Ole Maaloes Vej 5, DK 2200 Copenhagen, Denmark

^9^Genomics, Lawrence Berkeley National Laboratory, 84R01, 1 Cyclotron Road, Berkeley, CA 94720 USA.

^10^Mouse Informatics, EMBL-EBI, Wellcome Trust Genome Campus, Hinxton, Cambridgeshire, CB10 1SD, UK.

^11^Computational Bioscience Research Center, King Abdullah University of Science and Technology (KAUST), Ibn Al-Haytham Building -2, Thuwal 23955-6900, Kingdom of Saudi Arabia.

^12^Department of Biostatistics, Harvard School of Public Health, 655 Huntington Ave, Boston, MA 02115, USA.

^13^MRC Human Genetics Unit, MRC Institute of Genetics and Molecular Medicine (MRC-IGMM), University of Edinburgh, Western General Hospital, Crewe Road, Edinburgh, EH4 2XU, UK.

^14^Department of Cell and Molecular Biology, Karolinska Institutet, P.O. Box 285, SE-171 77 Stockholm, Sweden.

^15^Department of Clinical Genetics, VU University Medical Center Amsterdam, Van der Boechorststraat 7, 1081 BT Amsterdam, The Netherlands.

^16^Graduate Program in Areas of Basic and Applied Biology, Abel Salazar Biomedical Sciences Institute, University of Porto, Rua de Jorge Viterbo Ferreira n. 228, 4050-313 Porto, Portugal.

^17^Predictive Models for Biomedicine and Environment, Fondazione Bruno Kessler, via Sommarive 18, 38123 Trento TN, Italy.

^18^Department of Medicine, Karolinska Institutet at Karolinska University Hospital, Huddinge, SE-141 86 Huddinge, Sweden.

^19^Department of Dermatology and Allergy, Charité Campus Mitte, Universitätsmedizin Berlin, Chariteplatz 1, 10117 Berlin, Germany.

^20^Biozentrum, University of Basel, Klingelbergstrasse 50-70, 4056 Basel, Switzerland.

^21^Australian Institute for Bioengineering and Nanotechnology (AIBN), University of Queensland, Brisbane St Lucia, QLD 4072, Australia.

^22^Australian Infectious Diseases Research Centre (AID), University of Queensland, Brisbane St Lucia, QLD 4072, Australia.

^23^Department of Biological Sciences, University of Delaware, Newark, Delaware, USA.

^24^Bioinformatics and Computational Biology, The Jackson Laboratory, 600 Main Street, Bar Harbor, ME 04609 USA.

^25^Diamantina Institute, University of Queensland, Brisbane St Lucia, QLD 4072, Australia.

^26^Fondazione Santa Lucia, Dulbecco Telethon Institute, via del Fosso di Fiorano 64, 00143 Rome RM, Italy.

^27^Immunology and Infectious Disease, International Centre for Genetic Engineering & Biotechnology (ICGEB) Cape Town component, Anzio Road, Observatory 7925, Cape Town, South Africa.

^28^Division of Immunology, Institute of Infectious Diseases and Molecular Medicine (IIDMM), University of Cape Town, Anzio Road, Observatory 7925, Cape Town, South Africa.

^29^Department of Biochemistry and Mol. Biophysics, Columbia University Medical Center, 701 West 168th Street, New York, NY 10032 USA.

^30^Department of Biomedical Informatics, Columbia University Medical Center, 622 West 168th St. VC5, New York, NY 10032 USA.

^31^Institute of Cancer Genetics, Columbia University Medical Center, Herbert Irving Comprehensive Cancer Center, 1130 St. Nicholas Ave, New York, NY 10032 USA.

^32^Columbia Initiative in Systems Biology, Columbia University Medical Center, Herbert Irving Comprehensive Cancer Center, 1130 St. Nicholas Ave, New York, NY 10032 USA.

^33^Biological and Environmental Sciences and Engineering Division, King Abdullah University of Science and Technology (KAUST), Ibn Al-Haytham Building -2, Thuwal 23955-6900, Kingdom of Saudi Arabia.

^34^Applied Mathematics and Computational Science Program, King Abdullah University of Science and Technology (KAUST), Thuwal 23955-6900, Kingdom of Saudi Arabia.

^35^Laboratorio Nazionale del Consorzio Interuniversitario per le Biotecnologie (LNCIB), Padriciano 99, 34149 Trieste TS, Italy.

^36^Hubrecht Institute, Uppsalalaan 8, 3584 CT Utrecht, The Netherlands.

^37^The Royal Netherlands Academy of Arts and Sciences, P.O. Box 19121, NL-1000 GC Amsterdam, The Netherlands.

^38^University Medical Centre Utrecht, Postbus 85500 3508 GA Utrecht, The Netherlands.

^39^Genomics, Cold Spring Harbor Laboratory, 1 Bungtown Road, Cold Spring Harbor , NY 11797, USA.

^40^S..

^41^Institute of Pharmaceutical Sciences, ETH Zurich, Wolfgang-Pauli-Strasse 10, HCI H 303, 8093 Zurich, Switzerland.

^42^Department of Neurology, University at Buffalo School of Medicine and Biomedical Sciences, New York State Center of Excellence in Bioinformatics and Life Sciences, 701 Ellicott Street, Buffalo, NY 14203 USA.

^43^Gastroenterology, Research Center for Hepatitis and Immunology Research Institute, National Center for Global Health and Medicine, 1-7-1 Kohnodai, Ichikawa, Chiba, 272-8516 Japan.

^44^Department of Cancer Research and Molecular Medicine, Norwegian University of Science and Technology (NTNU), P.O. Box 8905, NO-7491 Trondheim, Norway.

^45^Department of Otology and Laryngology, Harvard Medical School, Massachusetts Eye and Ear Infirmary, Eaton-Peabody Lab, 243 Charles Street, Boston, MA 02114, USA.

^46^Department of Biosciences and Nutrition, Center for Biosciences, Karolinska Institutet, Hälsovägen 7-9, SE-141 83 Huddinge, Sweden.

^47^RIKEN Research Center for Allergy and Immunology (RCAI), 1-7-22 Suehiro, Tsurumi, Yokohama, Kanagawa, 230-0045, Japan.

^48^RIKEN Center for Developmental Biology (CDB), 2-2-3 Minatojima-minamimachi, Chuo-ku, Kobe, Hyogo, 650-0047 Japan.

^49^FM Kirby Neurobiology Center, Children’s Hospital Boston, Harvard . Medical School, 300 Longwood Ave, Boston MA 02115, USA.

^50^Department of Computer Science, University of Bristol, Merchant Venturers Building, Woodland Road, Clifton BS8 1UB, UK.

^51^Department of Biochemistry and Cell Biology, Rice University, Houston, Texas, USA.

^52^Cancer Biology Program, Mater Medical Research Institute, Raymond Tce, South Brisbane, QLD 4101, Australia.

^53^Department of Oncology, Division of Oncology, Biostatistics and Bioinformatics, Johns Hopkins University School of Medicine, 550 N Broadway, Baltimore, MD 21205, USA.

^54^Laboratory of Bioinformatics, Research Institute of Genetics and Selection of Industrial Microorganisms, 1-st Dorozhniy pr., 1, 117545 Moscow, Russia.

^55^Computational Biology Research Center, National Institute of Advanced Industrial Science and Technology (AIST), 2-4-7 Aomi, Koto-ku, Tokyo, 135-0064 Japan.

^56^Department of Medical Biochemistry, Tohoku University Graduate School of Medicine, 2-1 Seiryo-machi, Aoba-ku, Sendai, Miyagi, 980-8575 Japan.

^57^Department of Microbiology and Immunology, Keio University School of Medicine, 35 Shinanomachi, Shinjuku, Tokyo, 160-8582 Japan.

^58^Experimental Immunology, Academic Medical Center - University of Amsterdam, Meibergdreef 9 , 1105 AZ Amsterdam, The Netherlands.

^59^Department of Human Genetics, Leiden University Medical Center, Einthovenweg 20, 2333 ZC Leiden, The Netherlands.

^60^Department of Medical Genetics, Centre for Molecular Medicine and Therapeutics, Child and Family Research Institute , University of British Columbia , 950 West 28th Avenue, Vancouver, BC V5Z 4H4, Canada.

^61^Neuroscience, SISSA, via Bonomea 265, 34136 Trieste TS, Italy.

^62^Experimental Immunology, Immunology Frontier Research Center, Osaka University, 3-1 Yamadaoka, Suita, Osaka, 565-0871 Japan.

^63^RIKEN Advanced Science Institute (ASI), 2-1 Hirosawa, Wako, Saitama, 351-0198 Japan.

^64^Melanoma Research Center, The Wistar Institute, 3601 Spruce Street, Philadelphia, PA 19104, USA.

^65^RIKEN Bioinformatics And Systems Engineering Division (BASE), 1-7-22 Suehiro, Tsurumi, Yokohama, Kanagawa, 230-0045, Japan.

^66^Center for Molecular Medicine and Genetics, Wayne State University, 3228 Scott Hall, 540 East Canfield Street, Detroit, MI 48201-1928, USA.

^67^Laboratory Animal Research Center, Institute of Medical Science, The University of Tokyo, 4-6-1 Shirokanedai, Minato-ku, Tokyo, 108-8639 Japan.

^68^Science for Life Laboratory, Box 1031, SE-171 21 Solna, Sweden.

^69^Centre for Vascular Research, University of New South Wales, Sydney NSW 2052, Australia.

^70^Department of Hematopoietic Factor, Institute of Medical Science, University of Tokyo, Tokyo 108-8639, Japan.

^71^Western Australian Institute for Medical Research and Centre for Medical Research, The University of Western Australia, L6 MRF Building, Rear 50 Murray Street, Perth, WA 6000, Australia.

^72^Respiratory Medicine, University of Nottingham, Clinical Sciences Building, City Hospital, Hucknall Road, Nottingham, NG5 1PB, UK.

^73^Dermatology, School of Medicine, Kyungpook National University, 130 Dongdeok-ro Jung-gu, Daegu 700-721, South Korea.

^74^National Centre for Adult Stem Cell Research, Eskitis Institute for Cell and Molecular Therapies, Griffith University, Brisbane, Queensland, Australia.

^75^Systems and Computational Biology, Albert Einstein College of Medicine, 1300 Morris Park Ave Price 253, New York, NY 10461, USA.

^76^Division of Functional Genomics and Systems Medicine, Research Center for Genomic Medicine, Saitama Medical University, 1397-1 Yamane, Hidaka, Saitama, 350-1241 Japan.

^77^Faculty of Engneering, University of Bristol, Merchant Venturers Building, Woodland Road, Clifton BS8 1UB, UK.

^78^PRESTO, Japanese Science and Technology Agency (JST), 7 Gobancho, Chiyodaku, Tokyo, 102-0076 Japan.

^79^Center for Radioisotope Sciences, Tohoku University Graduate School of Medicine, 2-1 Seiryo-machi, Aoba-ku, Sendai, Miyagi, 980-8575 Japan.

^80^Anatomy and Embryology, Leiden University Medical Center, Einthovenweg 20, P.O. Box 9600, 2300 RC Leiden, The Netherlands.

^81^Division of Translational Research, Research Center for Genomic Medicine, Saitama Medical University, 1397-1 Yamane, Hidaka, Saitama, 350-1241 Japan.

^82^RIKEN BioResource Center (BRC), Koyadai 3-1-1, Tsukuba, Ibaraki, 305-0074 Japan.

^83^Department of Clinical Molecular Genetics, School of Pharmacy, Tokyo University of Pharmacy and Life Sciences, 1432-1 Horinouchi, Hachioji, Tokyo 192-0392, Japan.

^84^Department of Bioinformatics, Medical Research Institute, Tokyo Medical and Dental University, 1-5-45 Yushima, Bunkyo-ku, Tokyo, 113-8510 Japan.

^85^Department of Biochemistry, Ohu University School of Pharmaceutical Sciences, Misumido 31-1, Tomitamachi, Koriyama, Fukushima, 963-8611 Japan.

^86^Hjelt Institute, Department of Forensic Medicine, University of Helsinki, Kytosuontie 11, 003000 Helsinki, Finland.

^87^Department of Orthopedic, Trauma and Reconstructive Surgery, Charité Universitätsmedizin Berlin, Garystrasse 5, 14195 Berlin, Germany.

^88^Center for Clinical and Translational Reseach, Kyushu University Hospital, Station for Collaborative Research1 4F, 3-1-1 Maidashi, Higashi-Ku, Fukuoka, 812-8582 Japan.

^89^Netherlands Bioinformatics Centre, 260 NBIC, P.O. Box 9101, 6500 HB Nijmegen, The Netherlands.

^90^Graduate School of Pharmaceutical Sciences, Nagoya University, Furo-cho, Chikusa, Nagoya, Aichi, 464-8601 Japan.

^91^Department of Molecular and Cellular Biology, Harvard University, 16 Divinity Ave, Cambridge, MA 02138, USA.

^92^Department of Biochemistry, Nihon University School of Dentistry, 1-8-13, Kanda-Surugadai, Chiyoda-ku, Tokyo, 101-8310 Japan.

^93^Molecular and Cellular Oncogenesis, The Wistar Institute, 3601 Spruce Street, Philadelphia, PA 19104, USA.

^94^Department of Informatics, University of Bergen, Høgteknologisenteret, Thormøhlensgate 53, NO-5008 Bergen, Norway.

^95^Department of Biological and Medical Physics, Moscow Institute of Physics and Technology (MIPT) 9, Institutsky Per., Dolgoprudny, Moscow Region 141700, Russia.

^96^Current address: Massey University, New Zealand.

^97^RIKEN Preventive Medicine and Diagnosis Innovation Program

^98^RIKEN Center for Life Science Technologies (Division of Genomic Technologies)

^99^Regensburg Centre for Interventional Immunology (RCI), D-93042 Regensburg, Germany
